# Tumor-Associated Antigen xCT and Mutant-p53 as Molecular Targets for New Combinatorial Antitumor Strategies

**DOI:** 10.3390/cells10010108

**Published:** 2021-01-08

**Authors:** Jolanda Magri, Alessandro Gasparetto, Laura Conti, Enzo Calautti, Chiara Cossu, Roberto Ruiu, Giuseppina Barutello, Federica Cavallo

**Affiliations:** Molecular Biotechnology Center, Department of Molecular Biotechnology and Health Sciences, University of Turin, 10126 Turin, Italy; jolanda.magri@unito.it (J.M.); alessandro.gaspar489@edu.unito.it (A.G.); laura.conti@unito.it (L.C.); vincenzo.calautti@unito.it (E.C.); chiara.cossu@edu.unito.it (C.C.); roberto.ruiu@unito.it (R.R.)

**Keywords:** xCT, p53, PRIMA-1, APR-246, breast cancer

## Abstract

The cystine/glutamate antiporter xCT is a tumor-associated antigen that has been newly identified in many cancer types. By participating in glutathione biosynthesis, xCT protects cancer cells from oxidative stress conditions and ferroptosis, and contributes to metabolic reprogramming, thus promoting tumor progression and chemoresistance. Moreover, xCT is overexpressed in cancer stem cells. These features render xCT a promising target for cancer therapy, as has been widely reported in the literature and in our work on its immunotargeting. Interestingly, studies on the TP53 gene have revealed that both wild-type and mutant p53 induce the post-transcriptional down modulation of xCT, contributing to ferroptosis. Moreover, APR-246, a small molecule drug that can restore wild-type p53 function in cancer cells, has been described as an indirect modulator of xCT expression in tumors with mutant p53 accumulation, and is thus a promising drug to use in combination with xCT inhibition. This review summarizes the current knowledge of xCT and its regulation by p53, with a focus on the crosstalk of these two molecules in ferroptosis, and also considers some possible combinatorial strategies that can make use of APR-246 treatment in combination with anti-xCT immunotargeting.

## 1. Introduction

Cancer is one of the leading causes of death in the world. Although cancer therapy has improved in the past few decades, therapy failure is still common, resulting in poor patient outcomes. Therefore, new molecular targets and strategies to enhance therapeutic efficacy in patients with aggressive or resistant cancers are needed. Recently, the role exerted by redox homeostasis in the alteration of cancer metabolism, a hallmark of malignant progression and resistance to therapy, has suggested that the proteins involved in the regulation of redox balance may be promising therapeutic targets [1]. Indeed, cancer cells are characterized by enhanced reactive oxygen species (ROS) levels, which can sustain their proliferation rate and promote DNA damage and genomic instability. However, higher ROS levels can have detrimental effects on cancer cell survival, and are therefore compensated for through an increase in antioxidant mechanisms [2]. Interestingly, several genetic alterations, such as mutations in oncogenes and oncosuppressors, contribute to the dysregulation of cancer metabolism and redox homeostasis, providing a link between cancer genetics and metabolism [3].

In light of these considerations, xCT, which is the functional subunit of the cystine/glutamate antiporter system xc-, is a promising therapeutic target. Indeed, xCT is overexpressed by tumors of various histotypes, including breast and colorectal cancer, two of the most common cancer types, and regulates cell invasiveness and resistance to conventional treatments by contributing to the maintenance of intracellular glutathione (GSH) reserves, thus protecting against excessive ROS accumulation [4]. Interestingly, xCT expression is regulated by both wild-type and mutant p53 (mut-p53) [5], and its function is controlled by several growth factors and by PI3K [6,7]. Thus, xCT is a key player in the cross-talk between oncogenic or oncosuppressor pathways, redox balance and cell metabolism [4]. In particular, xCT decreases cancer cell sensitivity to small molecule inhibitors of mut-p53, such as APR-246, which binds and depletes GSH in cancer cells, triggering ferroptosis [5]. Indeed, high levels of xCT expression hinder this mechanism by restoring GSH pools, thus protecting from lipid peroxidation. This evidence suggests that, in mut-p53 tumors, xCT inhibition may synergize with APR-246 to induce cancer cell death.

In this review, we will summarize the effects exerted on cancer cells by xCT and both wild type and mut-p53, discuss their reciprocal regulation and speculate as to possible combined therapeutic strategies that can improve cancer treatment.

## 2. The Cystine/Glutamate Antiporter xCT

**Figure 1:**
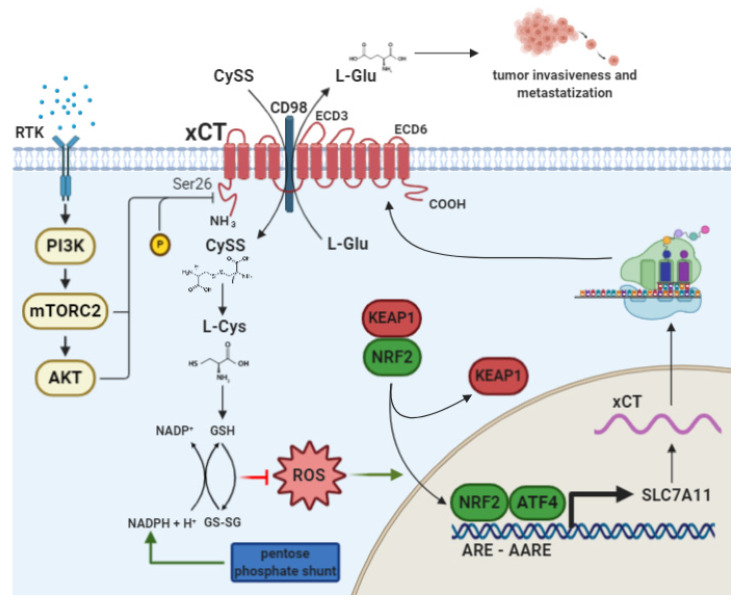
xCT function and transcriptional regulation. The cystine/glutamate antiporter xCT exports intracellular glutamate and imports extracellular cystine. Exported glutamate is involved in tumor microenvironment shaping, promoting invasiveness and metastasization. The imported cystine is reduced to cysteine and used for the biosynthesis of GSH, which is responsible for cancer cell protection from reactive oxygen species (ROS) excess due to genomic instability, altered metabolism and external insults, such as chemotherapy. ROS induce the upregulation of xCT, which destabilizes the KEAP1-NRF2 complex; NRF2 translocation into the nucleus and its binding to the ARE sequence on the *SLC7A11* gene promoter lead to xCT mRNA transcription and protein production. Amino acid depletion also induces xCT upregulation through ATF4, which binds to the Amino Acid Response Element (AARE) sequences on the *SLC7A11* gene promoter. The mTORC2/AKT signaling axis is another relevant pathway in xCT post-translational modulation that, when activated, inhibits xCT through Ser26 phosphorylation. This figure was created with BioRender.com.

The antiporter xCT is a 12-multipass transmembrane protein (Figure 1) encoded by the gene Solute Carrier Family 7, member 11 (*SLC7A11*). In association with the chaperone protein 4F2 heavy chain (4F2hc/CD98) encoded by the SLC3A2 gene, xCT forms the heterodimeric amino acid transport system xc- [8].

While 4F2hc is a promiscuous subunit for several amino acid transporters, xCT is specific for the activity of the system xc-.

xCT acts by exporting intracellular glutamate in exchange for extracellular cystine in a 1:1 ratio [8]. Once inside the cell, cystine is reduced to cysteine, which is involved as a rate-limiting precursor in the biosynthesis of the antioxidant molecule GSH [9]. Thus, xCT plays an important role in ROS detoxification. Indeed, high ROS levels can be harmful and cause oxidative damage to lipids, proteins and DNA, resulting in cellular alterations and possibly cell death [10]. Although xCT has a restricted expression pattern, limited to the brain, immune system and eyes, under physiological conditions [8], in cancer cells derived from most tumor types, the transporter is abundantly expressed at the cell membrane, where it promotes cell survival under oxidative stress conditions [8]. These functions are mostly due to the transcriptional upregulation of xCT expression, as mediated by the oxidative stress-responsive transcription factor NF-E2-related factor 2 (NRF2) and activating transcription factor 4 (ATF4) (Figure 1) [11,12]. However, recent studies have attributed more complex roles, beyond the regulation of redox homeostasis, to xCT, and have thus underscored its involvement in cancer progression, metabolism, chemoresistance and cancer stem cell (CSC) biology. These features and the transcriptional regulation of xCT will be briefly discussed in the following sections.

### 2.1. xCT Transcriptional Regulation by NRF2 and ATF4

The transcription factors mainly involved in xCT expression are NRF2 and ATF4, which are activated in response to stimuli such as oxidative stress and amino acid deprivation. Indeed, both NRF2 and ATF4 bind to cis-acting elements on xCT DNA, designated as the Antioxidant Response Element (ARE), that are also known as the Electrophile Response Element (EpRE), and Amino Acid Response Element (AARE), respectively. In particular, one ARE and two AAREs have been found in the mouse Slc7a11 promoter and these sequences are also conserved in the human SLC7A11 gene [11,12,13].

NRF2 is considered to be the master regulator of antioxidant defenses, since it regulates the expression of the genes involved in redox balance, detoxifying processes and metabolic reprogramming in cancer cells [14]. 

Treatment with electrophiles, such as diethyl maleate (DEM) which causes GSH depletion, lead to determine an increase in xCT mRNA levels in an NRF2-dependent manner [11]. According to this, the same treatment of NRF2-deficient cells causes profound cell damage [11]. The major regulator of NRF2 is Kelch-like ECH-associated protein 1 (KEAP1), which binds NRF2 with the Cullin-3 (CUL3)-ringbox protein 1 (RBX1) E3 ligase, mediating its ubiquitination and proteasomal degradation. Under pro-oxidative conditions, this complex is altered and NRF2 can translocate into the nucleus, activating target genes [14]. The treatment of human breast cancer cells with H_2_O_2_ causes an increase in the NRF2 nuclear fraction, and in xCT mRNA and protein levels [14]. On the other hand, the overexpression of the negative NRF2 regulator KEAP1 leads to the attenuation of SLC7A11 promoter activity, followed by reduced xCT protein levels [14]. 

Under stress conditions and amino acid deprivation, ATF4 is translated through an integrated stress response (ISR) dependent or independent pathway, thereby regulating the expression of genes involved in amino acid synthesis, differentiation, angiogenesis, metastatization, and drug resistance [14,15,16]. The tumorigenic functions of ATF4 are further confirmed by its overexpression in glioma cells, which induces high levels of xCT expression leading to an increase in glutamate secretion [16]. 

Furthermore, the deprivation of amino acids, such as cysteine and arginine, in mouse embryonic fibroblasts, induces high xCT mRNA expression levels, and the same effect is observed during endoplasmic reticulum (ER) stress [12]. This induction is due to the binding of ATF4 on the AAREs sequences in the xCT gene promoter. Indeed, in ATF4-deficient cells, system xc- activity is impaired [12] and their in vitro growth requires the medium to be supplemented with either cysteine or β-mercaptoethanol. Specifically, β-mercaptoethanol reduces cystine to cysteine and allows its uptake by other non-specific amino acidic transporters, thereby overcoming the requirement for xCT to replenish intracellular cysteine stores.

### 2.2. xCT as a Player in Cancer Cell Metabolism and Tumor Progression

xCT overexpression in many cancer histotypes suggests that it may have other key roles in cancer survival and progression beyond its well documented function in maintaining redox balance and in counteracting oxidative damage through GSH synthesis.

Studies in a number of glioma cell lines have reported that the expression of xCT and the regulatory subunit 4F2hc causes glutamate release, which can activate glutamate receptors, such as the ionotropic α-amino-3-hydroxyl-5-methyl-4-isoxazole-propionate (AMPA) receptor, in an autocrine or paracrine manner, causing glioma cell invasion and peritumoral excitotoxic neuronal cell loss [17]. More recently, Dornier et al. [18] have shown that both invasive MMTV-PyMT breast tumor-derived cells and MDA-MB-231 human cells release more glutamate than normal breast epithelial cells thanks to the acquisition of xCT expression. Furthermore, it has been demonstrated that the invasive protrusions observed in these cells depend on the presence of glutamine but not glucose in the culture medium, whereas glutamine deprivation reduces protrusion length. This indicates that glutaminolysis is responsible for invasiveness. Glutamate release activates the metabotropic glutamate receptor (GRM) that is expressed on cancer cells, which promotes Rab27 GTPase activity, which, in turn, mediates the trafficking of MT1-MMP metalloprotease on the cell membrane. The pharmacological inhibition of xCT with sulfasalazine (SAS) in vitro [17,18] and in vivo [17], via siRNA knockdown [18], or via the inhibition of the glutamate receptors or their downstream effectors [17,18], reduces the migration and invasiveness of cancer cells both in breast and glioma cancer cells. Further evidence for the involvement of xCT in metastasis formation comes from studies on xCT^KO^ melanocytes, in which the caveolin 1/β-catenin pathway, which is responsible for cell–cell adhesion, is activated as a consequence of p38-mediated caveolin 1 upregulation after ROS accumulation. This promotes the translocation of β-catenin from the nucleus to the cell membrane [19], thus inhibiting metastasis. On the other hand, xCT overexpression in glioblastoma cells confers resistance to oxidative stress induction and favors anchorage-independent cell growth, rendering them more tumorigenic [20].

In light of its function as an amino acid transporter, it is not surprising that xCT is implicated in the metabolic reprogramming of cancer cells and adaptation to nutrient availability in the tumor microenvironment, and many studies have uncovered an unexpected weakness in cancer cells cultured under glucose deprivation conditions [21,22,23]. Indeed, this may appear logical if we suppose that the effects of glucose starvation can be prevented by xCT’s detoxification of induced ROS. However, it has been demonstrated in several cell lines that xCT activity is responsible for glucose starvation-induced cell death [21,22,23]. In fact, xCT expression causes cell death upon glucose withdrawal, rendering cancer cells glucose-addicted. These effects are rescued when xCT is downregulated or pharmacologically inhibited with SAS. xCT amino acid transport, and not its antioxidant function, seems to be responsible for cell death upon glucose starvation, since xCT upregulation occurs independently of ROS generation under these conditions [22]. However, it is still unclear whether cystine uptake or glutamate export is the main cause of glucose addiction in cancer cells with elevated xCT expression. Indeed, both glucose and glutamine are considered to be important nutrients for cancer cell growth [22,23]. When glucose is depleted, cancer cells become more dependent on glutamine, which is converted to glutamate to produce α-ketoglutarate (α-KG), a key intermediate in the tricarboxylic acid (TCA) cycle, which generates substrates for oxidative phosphorylation (OXPHOS) to produce energy. If an excess of glutamate is exported due to xCT overexpression, the levels of α-KG drop, and a concomitant shortage of glucose leads to the collapse of the TCA cycle and mitochondrial respiration. Moreover, the increased xCT activity triggers increased nicotinamide adenine dinucleotide phosphate (NADPH) consumption in the reduction of cystine to cysteine. Upon glucose deprivation, the pentose phosphate pathway is blunted, and NADPH cannot be efficiently regenerated. This unbalance between NADPH consumption and regeneration ultimately leads to an impairment in the capacity to buffer cellular ROS [24]. This has been confirmed in all of the cited studies, as the addition of α-KG greatly improved cell viability under glucose starvation conditions [21,22,23]. interestingly, xCT expression during glucose withdrawal is dependent on NRF2 and ATF4 activation [22,23].

Cell metabolism reprogramming can make xCT-expressing cancer cells dependent on glutamine, as demonstrated in Non-Small Cell Lung Cancer (NSCLC) cells [25]. Indeed, xCT overexpression leads to higher consumption of cystine and other amino acids, and significant glutamate release. xCT-overexpressing lung cancer cells are more sensitive to glutamine withdrawal compared to xCT-knockdown cells and show a less invasive phenotype in the absence of glutamine, indicating that glutaminolysis becomes an essential metabolic pathway in the presence of high xCT levels. In general, these cells show higher glucose consumption as well as higher glutamine and lactate production, with the upregulation of OXPHOS, all hallmarks of metabolic cancer cell reprogramming [26].

Finally, the altered metabolic state caused by xCT expression also has consequences on mitochondrial functions, with mitochondrial genes being upregulated because of the increased OXPHOS [20]. Overall, these studies provide evidence that supports the key role played by xCT in both redox homeostasis and nutrient dependency in cancer cells, as discussed in detail by Koppula et al. [27].

### 2.3. xCT as a Player in Chemoresistance

During cancer development and progression, malignant cells show increased ROS levels, although the precise mechanisms leading to oxidative stress accumulation are still unclear [10].

Even though further oxidative insults induced by exogenous agents, such as chemotherapy, might render them more vulnerable to damage, cancer cells adapt well and develop enhanced endogenous antioxidant capacity through a set of adaptive mechanisms that may involve xCT, and thus redox homeostasis is maintained [10]. Recent evidence suggests that this adaptation contributes to malignant transformation, metastasis and resistance to anticancer drugs [10]. In particular, a correlation analysis on the National Cancer Institute’s 60 cell line panel has revealed that xCT expression negatively correlates with sensitivity to the compounds associated with a GSH-mediated resistance mechanism, suggesting that xCT expression induces chemoresistance through its GSH-mediated ROS detoxification activity [28]. Indeed, many studies have demonstrated that the combination of xCT targeting and chemotherapy can overcome such behavior. For example, the inhibition of xCT by SAS in human breast cancer cells leads to a decrease in GSH content, which is involved in drug detoxification and elimination, as it combines with anticancer drugs to mediate their export from cells by multidrug resistance proteins. Thus, this inhibition sensitizes cells to doxorubicin [29]. Similarly, the expression of xCT together with its stabilizer CD44v9 in human hepatocellular carcinoma cell lines is responsible for resistance to cisplatin, which is overcome by SAS treatment [30]. In glioblastoma, the pharmacological inhibition or silencing of xCT produces the same effect [20,31]. Moreover, erastin and sorafenib, two other drugs that are recognized as xCT inhibitors, promote sensitization to temozolomide treatment inducing ferroptosis [31], a non-apoptotic form of cell death [32], which is discussed below in detail. xCT can also mediate resistance to proteasome inhibition as well as chemotherapy. For example, in bladder carcinoma cells, bortezomib treatment causes NRF2 and ATF4 expression, with subsequent xCT mRNA upregulation, and acquisition of resistance to proteasome inhibition [13].

### 2.4. xCT as an Advocate for CSCs

xCT has been found to be overexpressed in CSCs derived from many tumors [4]. Like normal stem cells, CSCs possess self-renewal abilities and are capable of sustaining cancer cell growth via differentiation, and of driving resistance to chemotherapy [33]. Indeed, current anticancer therapies are directed against the bulk population of cancer cells, while CSCs, which acquire different mutational statuses and thus contribute to tumor heterogeneity, can escape from chemotherapy, leading to tumor relapse [34]. This is in part due to the high capacity of CSCs to protect themselves from ROS and oxidative stress, and this is partially due to xCT overexpression [33,35].

For instance, our research group has observed the upregulation of xCT in HER2^+^ and triple negative breast cancer cells grown as tumorspheres [36] that are enriched in CSCs [37]. In addition, xCT immunotargeting, as described below, impairs tumor growth and metastasization. Other scientists have observed CSC-like properties in xCT-overexpressing glioblastoma cells [38] and have demonstrated that the stem cell marker CD44v plays a role in increasing xCT expression in lung cancer cells, thus contributing to cisplatin resistance [39].

### 2.5. xCT Targeting as an Anticancer Therapeutic Strategy

The crucial role that xCT plays across many of the hallmarks of cancer means that targeting it is an encouraging approach to impairing tumor growth and metastasis formation. Several compounds for xCT inhibition, which show diverse mechanisms of action that all culminate in blocking xCT function and inducing ROS accumulation and cell death, have been tested (Table 1).

SAS was indicated as a cystine-uptake inhibitor of xCT activity for the first time in 2001 [40]. SAS causes a reduction in intracellular GSH levels and consequently reduces cell proliferation thanks to cell death. In almost all of the studies cited in this review, the pharmacological inhibition of xCT was obtained, in several different cancer cells, via SAS administration both in vitro and in vivo, and this clearly demonstrated its involvement in cancer biology. However, SAS can be considered a specific inhibitor of xCT only when used in vitro. In fact, SAS is composed of a sulfonamide antibiotic, sulfapyridine (SPY), linked to aminosalicylic acid (5-ASA), a nonsteroidal anti-inflammatory drug. When it is orally administrated in vivo, the compound is divided in its two components, missing its anti-xCT activity [57].

Recent studies have identified erastin, a RAS oncogene selective lethal compound (RSL), as a xCT inhibitor, and this discovery led to the unearthing of a new form of cell death named ferroptosis, which bears morphological and molecular characteristics that are different from previously identified types of cell death [32]. The treatment of cancer cells with erastin promotes the same effects as SAS, impairing cellular antioxidant defenses against ROS accumulation. This is due to cystine-induced starvation upon xCT inhibition, since the addition of β-mercaptoethanol, which promotes cysteine uptake via an alternative pathway, can rescue the erastin-induced effects [32].

Sorafenib is a multi-kinase inhibitor that has already been approved in clinics for the treatment of renal cell carcinoma [45]. It is able to induce ferroptosis and synergizes with erastin, leading to cell death [45]. Indeed, the treatment of fibrosarcoma cells with sorafenib can induce ferroptosis, which is rescued by ferrostatin-1 (Fer-1) and deferoxamine (DFO), two compounds that can inhibit ferroptosis [45]. Interestingly, sorafenib-induced ferroptosis can be described as a consequence of xCT specific inhibition, since sorafenib-treated cells display lower glutamate release, GSH depletion and accumulation of lipid peroxides, which are all characteristics of ferroptotic cell death upon xCT function impairment [32,45].

It is interesting to note that cell sensitivity to xCT inhibitors seems to correlate with xCT expression levels, as has already been observed for some oncogenes such as HER2 [58]. Indeed, as a consequence of xCT overexpression, glioma cells become resistant to xCT inhibitors such as sorafenib and erastin, and these effects are reversed upon ATF4 knockdown [16]. Similarly, the overexpression of xCT can also induce resistance to SAS treatment [31]. This effect could be ascribed to the increased threshold dose required for effective xCT inhibition in xCT-overexpressing tumors, and may be overcome by treatments that induce a chronic inhibition of xCT function.

Other erastin analogues are described in [41], while other reported but poorly characterized xCT inhibitors include HG106 [59], Capsazepine [60] and Compound A [52]. Further non-specific inhibitors of xCT (i.e., glutamate) are reviewed in [8].

In this perspective, xCT immunotargeting, consisting of several vaccination strategies, is a promising approach that has been developed by our research group (Table 1) [4]. To this end, we have developed several vaccines that are based on plasmid DNA, virus-like particles (VLPs) and viral vectors. Initial results showed that antibodies, induced by anti-xCT DNA vaccination in mice, are able to recognize and target xCT, causing ROS accumulation and GSH decrease in breast CSCs. Moreover, these antibodies are able to impair tumor growth and metastasis formation and to synergize with the cytotoxic effects induced by doxorubicin [36]. More recently, a new immunotherapeutic approach, which involves a VLP-based vaccine that displays the 6th extracellular domain (ECD6) of xCT, has been developed [55]. This vaccine can induce a functional anti-xCT antibody response, reproducing the effect of ROS accumulation and GSH decrease in breast cancer cells that have previously been observed with xCT antibodies induced by DNA vaccination. Moreover, we have shown that T cells and, to a greater degree, NK cells are recruited in the tumor microenvironment. These NK cells in the immune infiltrate can mediate an antibody-dependent cellular cytotoxicity (ADCC) via the recognition of anti-xCT IgG2a antibodies induced by vaccination [55]. In a similar way, a newer VLP vaccine that displays the third extracellular domain of xCT (ECD3), whose sequence is longer than that of ECD6, resulting in a major oligoclonal antibody response, can neutralize xCT function and impair breast cancer cell proliferation and metastasization [61]. Finally, the bovine herpesvirus 4 (BoHV-4)-based anti-xCT vaccine, which exploits a safe viral vector that confers immunogenicity to tumor antigens, has proven to be effective in impairing lung metastasis and inducing T lymphocyte activation, anti-xCT antibody production and ADCC in mouse mammary cancer models [53]. It is worth noting that anti-xCT vaccination impairs metastasis formation in a spontaneous mouse model of HER2^+^ mammary cancer, and synergizes with HER2-targeted therapies [56].

All this evidence supports the concept that xCT plays a role in tumor progression and metastasization, and that it is involved in chemoresistance, cancer metabolic reprogramming and CSC survival. This makes xCT a suitable target for anti-cancer therapy, and also paves the way for the combination of anti-xCT therapy with other drugs.

## 3. p53: More than a Genome Guardian

**Figure 2:**
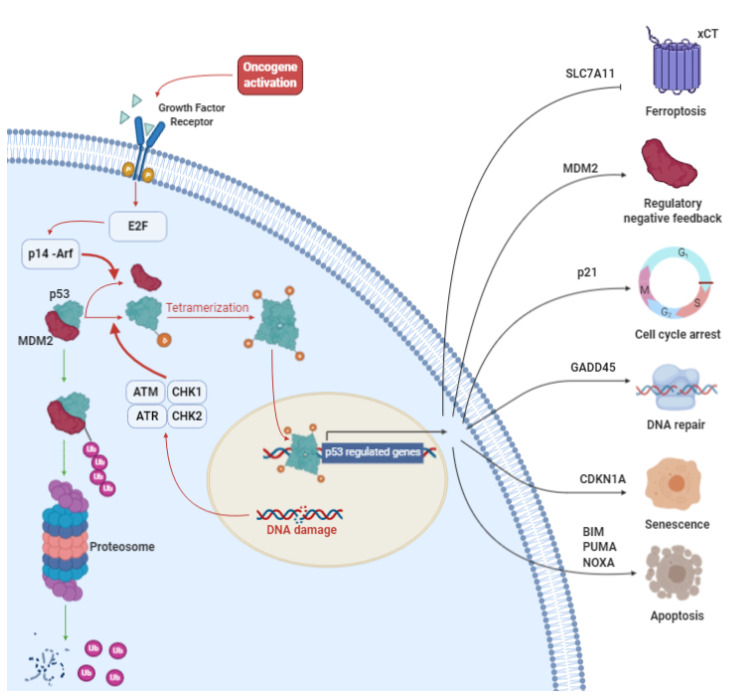
p53 function and transcriptional regulation. Under homeostatic conditions (green arrows), p53 is targeted by MDM2, which induces the proteasome-mediated degradation of p53. After cell injury or stress stimuli (red arrows), p53 levels increase due to several activated pathways that converge on the inhibition of MDM2 or post-translational p53 modifications (e.g., acetylation, phosphorylation). p53 activation strengthens the thermodynamic stability of the protein and induces its homo-tetramerization, inducing the transcription of target genes that are responsible for cell cycle arrest, DNA repair, senescence, apoptosis and ferroptosis. p53 is also a direct transcriptional inducer of MDM2, establishing a negative feedback loop. The role of p53 in inhibiting xCT gene transcription, thus contributing to ferroptosis, is also indicated. This figure was created with BioRender.com.

Cellular tumor antigen p53, often called the “guardian of the genome”, is an intracellular transcription factor encoded by the TP53 gene, which is located on the short arm of chromosome 17 [62]. p53 is the prototype tumor suppressor gene [63], as it plays an essential role in a large number of processes under both physiological and pathological conditions (Figure 2). Indeed, it regulates genomic stability [64], DNA repair [65], oxidative stress [66], metabolism [67], apoptosis [68,69], differentiation [70], cell cycle control, stemness, migration [71], metastasis [71], autophagy [70], angiogenesis [70], senescence [72], and drug sensitivity [73].

The central element of the p53 protein is the DNA-binding domain (DBD), which is shared by other p53 family members (e.g., p63, p73; for a review, see [74]), that allows the protein to bind response elements on its target genes. This region plays a fundamental role in p53 activity, and many mutations can occur within this sequence [70]. The N-terminal region of p53 is the transcription-activation domain (TA), which is the binding site for positive (e.g., p300/CBP) and negative (e.g., MDM2) regulators [75]. The C-terminal region contains the oligomerization domain (OD), where alternative splicing and post-translational modifications occur and influence the DNA binding ability and transcriptional activity of p53 [76].

TP53 is a housekeeping gene and its transcriptional product is continuously present inside the cells. However, under homeostatic conditions, cells contain very low levels of the p53 protein, despite high mRNA expression [77]. The main reason for this is that p53 is targeted by Mouse Double Minute 2 homolog (MDM2), a E3 ubiquitin ligase, which mediates p53-proteasomal degradation [78]. However, after cell injury (e.g., DNA damage by UV irradiation [79]) or stress stimuli, the concentration of p53 rises rapidly via several activated pathways that converge on the inhibition of MDM2 or post-translational p53 modifications (e.g., acetylation, phosphorylation) [80]. One of the most studied mechanisms and most frequently described p53 modifications is the activation of mutations in ataxia-telangiectasia (ATM) and A-T and Rad3-related (ATR) protein kinases, which mediate p53 phosphorylation on Ser15 (Ser18 in mouse) [81]. p53 activation makes it more thermodynamically stable and induces its homotetramerization [82]. When activated, p53 induces the transcription of p21, a cyclin-dependent kinase inhibitor that is crucial for p53-dependent cell cycle arrest at the G1/S phase and senescence. p53 activation is also an essential event in the G2/M cell cycle checkpoint (see [83] for review). Here, p53-induced cell cycle arrest makes it possible for cells to attempt to repair genomic damage. p53 itself induces the activation of genes involved in these processes (e.g., GADD45) [84]. If cells experience an important insult that the DNA-repairing mechanisms are not able to remediate, p53 leads cell to apoptosis or necrosis by triggering the expression of direct target genes, including the p53 upregulated modulator of apoptosis (PUMA), Phorbol-12-myristate-13-acetate-induced protein 1 (NOXA) and Bcl-2-like protein 11 (BIM). These proteins act by inhibiting the family of Bcl-2 anti-apoptotic proteins (e.g., BCL-2, Bcl-xL). In this context, pro-apoptotic elements such as Bcl-2-associated X protein (BAX) and Bcl-2 homologous antagonist killer (BAK) are free to induce the release of cytochrome C from mitochondria and activate the intrinsic apoptosis pathway [85]. Finally, p53 is also a direct transcriptional inductor of MDM2, establishing a negative feedback loop [86]. However, the action of p53 cannot be exclusively limited to a dichotomic activation of downstream pathways. Recent studies have shown that not all the activation signals of p53 are equal. Besides its role in response to DNA damage, which contributes to tumor suppression by either permitting DNA repair or by removing cells harboring potentially oncogenic alterations, its activation in many other conditions, including improper cell proliferation driven by oncogene activation, nutrient deprivation, telomere erosion, and hypoxia, has been investigated in recent years [87]. These signals do not activate p53 through the same pathways. For example, the p53 response to aberrant oncogene activation is primarily mediated by p14-ARF, a small protein that binds and inhibits MDM2, whereas p14-ARF does not appear to be involved in the p53-dependent response to DNA damage. Some researchers have investigated which of the ATM/ATR or p14-ARF pathways is more involved in tumor suppression. The answer is still unclear, and the results are contradictory [88,89]. These are only some starting and provocative suggestions to keep in mind when reconsidering the importance of p53 response to DNA damage in tumor suppression and the real role of p53 in cell economy.

### 3.1. p53 in Cancer

TP53 is the most mutated gene in human cancer [90], and, at some point during tumor progression, about 50% of cancers acquire a p53 mutation [91]. Modifications have been found in every region of the protein [90], but only some of the most frequently occurring mutations have seen their contribution to cancer progression thoroughly studied. While most tumor suppressor genes are inactivated by mutations, leading to loss of protein synthesis, missense mutations represent 80% of the alterations for TP53 [91]. Frameshift and nonsense mutations, which normally result in the loss of p53 expression, are possible in any case. Missense mutations consist of a single nucleotide substitution and may occur all along the TP53 gene, but most commonly involve the DNA binding region within six mutational hotspots (R175, G245, R248, R249, R273 and R282) [92]. These mutations generally induce a loss of or reduction in the wild-type activity of p53. Interestingly, since p53 normally acts as a tetramer, mut-p53 may also function as a dominant negative inhibitor of the remaining wild-type p53 [93]. Moreover, mutated-p53 (mut-p53) may show a gain of function (GOF) phenotype. This hypothesis is supported by several studies in both human and mouse cell lines, although it has been shown that this phenomenon has a tissue-dependent manifestation [93]. For example, Jackson et al. have shown how mut-p53 does not have GOF activity in K-Ras-initiated lung adenocarcinomas [94]. One important piece of evidence for GOF mutations is the observation that patients carrying a *TP53* germline missense mutation display significantly earlier cancer onset than patients with mutations that result in a loss of p53 protein expression [95]. Plentiful in vitro and in vivo experiments have confirmed the ability of mut-p53 to enhance invasion, migration, proliferation, colony formation, genomic instability, stemness, angiogenesis, epithelial–mesenchymal transition and drug sensitivity. In particular, these improved abilities depend on the mutation site and nucleotide substitution, and correlate with a worse prognosis [93]. As previously mentioned, p53 mutations occur preferentially in six hotspots. It has been shown that different nucleotide substitutions that occur in the same location may lead to different phenotypes. R273H, a common p53-mutation, improves cell survival and makes cells more resistant to drugs, whereas the R273C mutation, a different amino acid substitution in the same position, only improves cell survival without altering drug resistance [93].

As described by Muller and Vousden, mut-p53 essentially acts via four mechanisms, which inevitably overlap, that affect its DNA-binding ability and its interaction with transcription factors and other proteins [92]. One interesting mut-p53 impact is on the expression of NRF2 target-genes. Lisek et al. have shown that mut-p53 may differentially affect the expression of subsets of NRF2 targets, increasing the expression of a proteasome subunit gene (PSMC1), thioredoxin system genes (TXN, TXNRD1), and the Glutamate-cysteine ligase regulatory subunit *(GCLM)*, while downregulating targets such as HMOX1, SLC7A11 and ABCC3 [66]. Another interesting finding is that under some conditions, such as hypoxia or after serum stimulation, wt-p53 may behave like mut-p53 [96,97].

In conclusion, p53 is one of the most interesting cellular targets for limiting tumor growth while also translationally improving the duration and quality of a patient’s life.

### 3.2. Principles of p53 Targeted Therapy

Knowledge on the processes and molecular bases of p53 action in health and disease are far from being conclusive. Even its well-known involvement in carcinogenesis now appears to be more complex and less well understood than previously. Many questions are currently unanswered and the categorization of biological phenomena in strict classes may limit the full understanding of the intrinsic polymorphic nature of p53. However, what does currently appear certain is the proliferative advantage that mut-p53 gives to cancer cells, although it is unclear whether the p53 mutation plays a promoting or an initiating role [93]. Nevertheless, the importance of, and addiction to, p53 mutations in tumor cells is well known. Thanks to these fascinating implications, several strategies are attempting to target cancer cells that express mut-p53, and to activate wt-p53 in non-mutated cells. Several agents that are endowed with the ability to inhibit the activity of p53 negative modulators have been investigated as means to induce p53 activation in cells harboring wt-p53. Many studies have established the effect of these therapies and emphasized the potential of these inhibitors [98,99]. As seen above, the main, but not only, mechanism by which p53 is degraded is MDM2 binding. Nutlins are the first group of small molecules that have been investigated and found to act as wt-p53-MDM2 interaction inhibitors. These drugs bind the p53-binding site on MDM2 by mimicking the crucial amino acidic interaction. This MDM2 post-translational modification sterically inhibits interaction with p53 and induces p53 accumulation and the restoration of its transcriptional activity, followed by cell cycle arrest and apoptosis in MDM2-overexpressing tumor cells [73,100]. This group of small molecules is formed of three compounds, named nutlin-1, -2, -3. Combined therapies have been tested and the synergistic effects of nutlin-3 with cytostatic drugs have been reported (e.g., with cisplatin) [101]. However, although these small molecules have sufficient permeability to enter cells and elicit the dose-dependent accumulation of wt-p53, they are not able to induce cell cycle arrest or upregulate p53 downstream target genes in mut- or p53-null tumor cells [73]. Their poor capacity to have a therapeutic effect in cells carrying mut-p53 means that nutlins have a limited field of usability. Several other strategies have followed for the treatment of cells with mut-p53: restoring wt-p53 conformation, promoting mut-p53 degradation, and targeting mut-p53 regulated pathways [93]. In particular, APR-246 (PRIMA-1^met^), the methylated analogue of PRIMA-1 (p53-reactivation and induction of massive apoptosis-1), is the first mut-p53 reactivating compound to enter clinical trials in patients with hematologic and solid malignancies, such as gastric, bladder and NSCLC, that bear p53 mutations. Its safety was demonstrated in phase 1 and other ongoing clinical trials [102] (www.clinicaltrials.gov).

### 3.3. PRIMA-1 and Its Analogues

PRIMA-1 is a low-molecular-weight compound discovered in 2002 after a molecular screening of a library of 2000 molecules from the National Cancer Institute that was performed to identify compounds that are able to restore the wild-type function of mut-p53 and suppress the growth of human tumor cells in a p53–dependent manner. It was first reported by Bykov et al. [103], who called this new drug PRIMA-1, from “P53 Reactivation and Induction of Massive Apoptosis”. Since 2002, other analogues of PRIMA-1 have been developed, in particular PRIMA-1^Met^, named APR-246. This analogue was presented in 2005 and appears to be more active and have better permeability proprieties than its precursor [104,105]. The antitumor activity of this compound has been evaluated in several cancer cell lines, as extensively reviewed by Pendrix et al. [106]. PRIMA-1 and its analogues are pro-drugs that are rapidly activated after their administration, via conversion into methylene quinuclidinone (MQ), the active intermediate of the drug. Indeed, the antitumor activity of APR-246 has mainly been described as relating to mut-p53 reactivation due to the covalent binding of MQ to thiol groups on cysteine 124 and 277 residues in the core domain of mut-p53. This drives a conformational change resulting in the reactivation of p53 pro-apoptotic functions [107]. The administration of these drugs results in the refolding of p53 into the wild-type conformation and the activation of p53-downstream pathways. After reactivation, p53 moves to the nucleus and accumulates in the nucleoli [68,108,109]. The restoration of the transcriptional activity of mut-p53 has been tested by several groups in many cell lines. However, the findings are not conclusive. It is clear that APR-246 and its analogues are tumor-suppressor drugs that are able to activate intracellular-caspase-dependent apoptosis [106]. It is also clear that the transcriptional reactivation of p53-target genes is tissue- and p53-mutational state-dependent. However, the majority of researchers agree that these drugs induce the upregulation of p21, Bax, NOXA and PUMA (there is less consensus on the latter) [73,106]. It has also been shown that PRIMA-1 can upregulate microRNA-34a, a small non-coding RNA that is positively regulated by wt-p53, inducing apoptosis in cancer cells [110].

Interestingly, many researchers have noticed that PRIMA-1 and its analogue APR-246 have a cytotoxic effect even when administrated to p53-null and p53-knockdown cells [111,112]. Several studies have also investigated the influence of these drugs on the Unfolded Protein Response (UPR) and seems that they may interact with UPR by increasing the expression of HSP70, HSP90, XBP1, GRP78 and CHOP [106,113]. This is in line with the global consensus that PRIMA-1 and APR-246 induce an increase in the amount of ROS and decrease GSH cellular content via a decrease in antioxidant response and an increase in ROS production [5,104,106,114]. Indeed, MQ is able to covalently bind GSH and inhibit its recycling by GSH-reductase [5]. Moreover, treatment with PRIMA-1 or APR-246 leads to the downregulation or inhibition of antioxidant enzymes (e.g., TRXR1, PRX3, or GPX-1) [111,115,116]) and an alteration in the NFE2L2/HMOX1 axis [115]. On the other hand, APR-246 converts the TrxR1 enzyme into a NADPH oxidase, producing an increase in ROS amounts [115]. Interestingly, higher xCT expression makes cells less sensitive to the ROS-balance disruption caused by the treatment. Therefore, in this context of ROS-balance modulation, Liu et al. have proposed xCT as a predictive biomarker for APR-246 cellular sensitivity [5], suggesting the existence of crosstalk between xCT and p53, as discussed in detail in the following paragraphs. 

## 4. xCT and p53 Interplay in the Regulation of Ferroptosis

Ferroptosis has been described by Dixon et al. as a new form of cell death with specific features [32]. Fibrosarcoma HT-1080 cells treated with erastin show a mitochondrial deficit and the depletion of intracellular ATP, ROS accumulation, and lipid peroxidation. These effects are not modulated by inhibitors of apoptosis or necroptosis. Indeed, ferroptosis occurred in Bax/Bak deficient cells, indicating that it is biochemically distinct from these well-studied forms of cell death [32]. Moreover, the authors found that Fer-1 acts as a specific small molecule inhibitor of ferroptosis, preventing the erastin-induced accumulation of cytosolic and lipid ROS, and demonstrated that DFO, an iron chelator, can rescue erastin-induced cell death, suggesting that ferroptosis is an iron-dependent mechanism [32].

xCT has been found to participate in ferroptosis. Indeed, GSH depletion upon xCT inhibition by SAS or erastin leads to the iron-dependent accumulation of ROS, especially lipid ROS, leading to cell killing [32]. This depends on the impairment of GSH-dependent peroxidase 4 (GPX4) activity, which in normal conditions catalyzes the reduction of organic hydroperoxides to water or the corresponding alcohols, using GSH as an essential cofactor [47].

Recent findings have shown that erastin-induced ferroptosis triggers ER stress, dependent on the activation of the eIF2alpha-ATF4 branch of the ER stress response pathway, that can be upregulated by amino acid depletion [45]. The activation of this pathway is likely due to the intracellular cysteine depletion upon xCT inhibition by erastin. Indeed, upregulation of the ER stress-responsive genes downstream of ATF4 occurs upon system xc- inhibition.

Furthermore, beclin1 (BECN1), a key player of autophagy in the class 3 phosphatidylinositol 3-kinase (PtdIns3K) complex, directly binds to xCT after its phosphorylation at Serine 90/93/96 by AMPK. This complex inhibits xCT activity by promoting GSH depletion and lipid peroxidation following erastin or SAS treatment [117].

Another line of research has shed light on an unconventional effect that p53 activity has on xCT expression, and that results in the induction of ferroptosis. Compelling evidence of p53/SLC7A11 regulatory circuitry that is active in both normal and cancer cells has been provided by Jiang and colleagues, who demonstrated that the SLC7A11 gene is a target for p53-mediated transcriptional repression [118]. In particular, wild-type p53 inhibits the expression of SLC7A11 by binding to a consensus sequence located at 5′ to the transcription start site of the human gene. The authors have shown that erastin induces high levels of cell death in cells carrying wt-p53 and only low levels have been observed in p53-null cells. However, treatment with Fer-1 completely rescued wt-p53 cells from erastin-induced cell death [118]. By lowering SLC7A11 gene expression, p53 sensitizes cells to ferroptosis by reducing xCT-mediated cystine uptake, GSH production and consequent protection from oxidative stress. Interestingly, p53 binding at the SLC7A11 gene is not the only mechanism through which p53 modulates xCT expression and contributes to ferroptosis. The monoubiquitination of histone H2B at lysine 120 (H2Bub1), a key epigenetic modification in the regulation of gene transcription and chromatin organization, is needed for SLC7A11 expression and inhibits ferroptosis in unstressed cells. p53 may induce a decrease in the levels of H2Bub1 by promoting the nuclear translocation of deubiquitinase USP7, and thus contributes to the inactivation of SLC7A11 expression [119].

Ferroptosis participates significantly in p53 tumor suppression and relies on a mechanism that is independent of “canonical” p53 activities, since it can also be triggered by acetylation-defective mut-p53 that lacks the ability to induce growth arrest, senescence and apoptosis [118]. Although SLC7A11 gene expression is negatively affected by p53, it is increased by transcription factors engaged by oxidative stress such as NRF2 and ATF4 [13,14,16], while endogenous p53 levels are not strongly upregulated by cellular ROS [118]. xCT overexpression, in turn, restrains the ability of p53 to induce ferroptosis and to suppress tumor formation. However, robust increases in both ROS and p53 levels (the latter achieved via genetic or pharmacological manipulation), synergize in inducing ferroptosis in a synthetically lethal fashion. As recently reviewed by Liu and colleagues, wt-p53 may not only positively modulate ferroptosis, but it may also have an anti-ferroptotic role. Many processes are involved in this mechanism, including protein complexes and miRNA [120,121]. In summary, p53 has context-dependent activity and may play a dual role. The expression and activity of xCT may be one of the elements that dictate the outcome of p53 functions towards cell survival or death. Overall, that evidence provides a rationale for a combinatorial use of xCT inhibitors and p53 agonists in the management of wt-p53 tumors.

Moreover, even mut-p53 is involved in ferroptosis regulation. In particular, when p53 carries GOF missense mutations, it is able to bind NRF2, inducing the transcriptional inhibition of SLC7A11. Mut-p53 cells are therefore sensitized to ferroptosis and less resistant to oxidative-damage-inducing drugs [120]. This clears the way for the rational development of combinatorial therapeutic strategies for mut-p53 cancer cells.

## 5. xCT Targeting and APR-246 Administration: A New Therapeutic Combinatorial Strategy

At present, little is known about a combinatorial approach that includes xCT targeting and p53 reactivation through APR-246 treatment. The dual mechanism of action of APR-246, which includes a role in GSH and ROS modulation together with its effects on mut-p53, is, however, quite well established. In particular, the active component of APR-246 has been shown to react with GSH, forming an adduct (GSH-MQ) that depletes GSH from the cells, stopping its recycling by GSH reductase [5]. This leads to ROS accumulation, especially in mitochondria, causing lipid peroxidation, mitochondrial rupture and cytochrome-c release. In mut-p53 osteosarcoma cells, APR-246 can inhibit thioredoxin-1 (Trx1) and glutaredoxin-1 (Grx1), which are commonly upregulated in cancer cells, and favor their protection against oxidative stress [116]. Moreover, the treatment of breast cancer cells with APR-246 for 12 h causes perturbations in gene expression, with six genes involved in ROS modulation being differentially expressed in all of the cell lines investigated. SLC7A11 is among these genes [114]. Therefore, these results have revealed the dual role that APR-246 plays as a reactivator of mut-p53 and an inducer of ROS accumulation in cancer cells. This latter mechanism might explain why this drug can even exert anticancer activity in wt-p53 cells.

In the context of p53-mediated modulation over xCT expression and activity, Liu and colleagues have shown for the first time that xCT blockade and APR-246 administration may act synergistically to target and kill mut-p53 esophageal cancer cells [5]. As a result of APR-246 treatment, ROS levels increase and GSH cellular stores become eroded, and the attenuated xCT expression, resulting from mut-p53-mediated NRF2 inhibition, further reduces the ability of cancer cells to cope with oxidative stress. However, similarly to what was described by Jiang and colleagues in the context of wt-p53 [118], the actual levels of xCT expression are crucial for the sensitivity of mut-p53 cells to oxidative damage, and the expression level of xCT may serve as a predictive biomarker of the response of mut-p53 cancers to APR-246. In particular, cells with low xCT levels have proven to be the most sensitive to the treatment.

**Figure 3:**
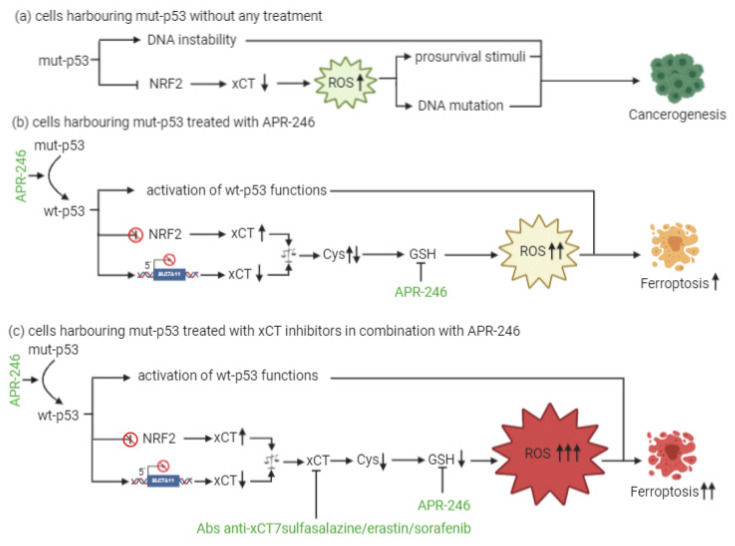
A schematic explanation of the rationale for combined treatment. (**a**) The effects of mut-p53 on cell cycle and redox balance. mut-p53 favors cancerogenesis by inhibiting NRF2-dependent xCT transcription and consequently slightly increasing the amount of intracellular ROS, which stimulates the cell to proliferate. (**b**) The effects of APR-246 treatment. mut-p53 is converted to its active form (wt-p53), leading to pro-apoptotic functions and the selective induction of ROS increase via GSH depletion, although the mut-p53-dependent inhibition of xCT expression is lost. This causes cells to undergo ferroptosis. (**c**) The effects of the combined treatment. APR-246-dependent GSH depletion together with xCT inhibition sharply decrease GSH levels, inducing high ROS levels that overwhelm the antioxidant capacity of the cell and finally lead to ferroptotic death. This figure was created with BioRender.com.

Remarkably, the therapeutic efficacy of APR-246 in mut-p53 harboring cancer cells can be sharply increased by concomitant xCT inhibition, leading to the synergistic induction of ROS accumulation and apoptosis [5]. The rationale for such a combinatorial strategy is further supported by the notion that APR-246 disrupts the interaction between mut-p53 and NRF2 [122]. Therefore, it has been proposed that this can release NRF2 from mut-p53 inhibition restoring elevated xCT levels, and potentially antagonizing the therapeutic activity of APR-246, thanks to GSH depletion [123]. Based on these findings, the increased therapeutic efficacy of the combination of APR-246 and xCT inhibitors is unlikely to be the result of the restoration of wt-p53 functions with respect to SLC7A11 gene expression, since wt-p53 should also lead to its transcriptional repression, albeit through a distinct molecular mechanism, but rather it appears to depend on APR-246 oxidative stress induction (Figure 3). Overall, these lines of evidence suggest that the predominant mechanism underlying APR-246 therapeutic activity relies on an impairment of cancer cell antioxidant defenses, an effect that can be dramatically augmented in the context of xCT inhibition.

## 6. Additional Potential Targets for Combined Therapies

One main concept emerging from the sections above is the fact that the inhibition of xCT functions is key for the optimal therapeutic response of mut-p53 tumors to APR-246. Therefore, therapeutic attempts that aim to cause at blockade of xCT functions may benefit from the concomitant modulation of endogenous signaling pathways that physiologically limit xCT activity. The mTORC2/AKT signaling axis is one signaling pathway that has recently emerged as a key negative regulator of xCT antiporter activity without necessarily altering its protein expression (Figure 1) [6,7].

In cancer cells, hyperactive receptor tyrosine kinases (RTK) and constitutively activated PI3K signaling result in the direct phosphorylation of xCT Ser26 by mTORC2 [7] and/or AKT [6], respectively. The amino acid sequence surrounding xCT Ser26 shares features that are common to both mTOR and AKT substrates, which possibly explains the promiscuous affinity of both mTORC2 and AKT for this phosphorylation site [6,7]. Whatever the identity of the direct upstream kinase, xCT antiporter activity is substantially impaired upon Ser26 phosphorylation. Pharmacological or genetic mTORC2 inhibition in cancer cells induces higher xCT activity and GSH synthesis [7], while cancer cells with hyperactive PI3K signaling become metabolically more vulnerable to methionine deprivation via the inhibition of xCT expression and function [6]. This is of particular clinical significance because PI3K and its downstream signaling are frequently hyperactivated in cancer patients, and this is considered a prominent oncogenic driver event per se [124,125]. Pharmacological strategies based on PI3K or mTOR inhibition as stand-alone therapies have substantially failed to achieve clinical benefits in most oncological settings, in part because of the significant toxic effects that are secondary to the systemic inhibition of the pathway, and in part because of acquired or intrinsic resistance mechanisms to PI3K/mTOR inhibition [126,127,128]. In this context, it seems possible that one mechanism of resistance to PI3K/mTOR inhibitors may be provided by the enhancement of xCT pro-survival, which are antioxidant functions caused by its curtailed Ser26 phosphorylation, ultimately rendering cancer cells less vulnerable to oxidative stress. For instance, in glioblastoma cells, mTORC2 deficiency enhances the ability of cancer cells to adapt to unfavorable environmental conditions [7]. Moreover, mTORC2-deficient epithelial cells are significantly more resistant to oxidative stress [129]. Therefore, it is tempting to speculate that mTORC2/AKT upstream agonists may synergize with xCT inhibition and APR-246 in the eradication of tumor cells.

## 7. Conclusions

Evidence from research and clinics has clarified that effective cancer treatment requires the simultaneous targeting or modulation of several pathways and biological functions that play key roles in cancer cell survival, such as oncogenic pathways, cell metabolism and redox balance. In this context, the crosstalk that occurs between mut-p53 GOF forms and the oxidative stress regulator xCT are a promising target for combination therapies. Indeed, mut-p53 inhibits the NRF2-dependent transcription of SLC7A11, leading to a low expression of xCT. This results in reduced GSH production and enhanced basal ROS levels compared with normal cells. In the absence of the wt-p53 tumor suppressor function, this increased oxidative stress may promote tumorigenesis through oxidative DNA damage and genomic instability [123]. Moreover, low levels of xCT, induced by its inhibitors, sensitize cancer cells to ferroptosis. This evidence has prompted us to reason that the restoration of wt-p53 function by APR-246 and its consequent activation in mut-p53 cells will lead to the canonical apoptotic pathways mediated by p53, and, in the meanwhile, that this intervention can restore cell sensitivity to xCT inhibition by increasing its expression. Therefore, the administration of APR-246 in combination with xCT targeting will bring cancer cells to death. In this light, the use of xCT-targeting vaccines, which determine a specific xCT inhibition, appears to be a more promising strategy than the use of synthetic inhibitors, since no drug compounds with specificity for xCT alone have been reported yet. Indeed, vaccination induces a durable immune response that exerts anti-cancer activities via different mechanisms, including the antibody-mediated inhibition of xCT function, which promotes ferroptosis, and the ADCC of xCT expressing cells [4]. Moreover, as discussed above, xCT has been found to be overexpressed in CSCs, contributing to drug resistance [4,33,35]. In a similar way, p53 GOF mutations have been reported in CSCs, determining de-differentiation of cancer cells and drug resistance [130]. These notions further support the rationale for a p53 and xCT dual targeting, which may potentially hit even stem cells among the tumor bulk population.

Of course, this combinatorial approach required further investigation. Moreover, extensive investigations into the potential interference of APR-246 treatment with the success of anti-xCT vaccination are required. Indeed, Zhang et al. have proposed targeting CSCs using APR-246 in combination with dendritic cell-based vaccination against p53. They demonstrated the efficacy of APR-246 in killing CSCs and inhibiting tumorsphere formation. However, they failed to observe the combined therapy having a significant effect on increasing tumor-free survival in a methylcholantrene-induced in vivo tumor model when compared with single treatments. They ascribed their unsuccessful data to the inadequacy of the cancer model chosen, and to the ability of APR-246 to decrease the activation of human and mouse immune cells [131]. If confirmed, this drawback can be easily overcome by a therapeutic protocol that provides anti-xCT vaccination prior to APR-246 administration, or by adjusting the APR-246 administration dose accordingly.

In conclusion, as already indicated by Liu et al. [5], we propose that the combination of xCT immunotargeting with drugs that can restore the function of mut-p53 may be a new opportunity for the treatment of patients suffering from cancers that bear p53 mutations. To this end, further studies are warranted to set up effective combination strategies and to evaluate the role that the other members of the p53 family—p63 and p73—might play in the regulation of xCT and its interplay with p53.

## Figures and Tables

**Table 1:** A list of xCT inhibitors.

Name	Molecular Targets Other than xCT	Pre-Clinical Use In Vivo	Current Clinical Indications
Sulfasalazine (SAS) [40]	NF-κB [41]	Yes (but limited by low solubility and poor bioavailability) [42]	Second-line treatment of ulcerative colitis [43]
Erastin [44,45]	VDAC2, VDAC3 [46]	No (poor metabolic stability and low solubility) [47,48]	None
Piperazine Erastin (PE) [47]	Not investigated	Yes (Erastin analogue with increased water solubility and metabolic stability) [47]	None
Erastin Ketone Imidazole (IKE) [48]	Not investigated	Yes (Erastin analogue with increased potency, selectivity, water solubility and metabolic stability) [48,49]	None
Sorafenib [45]	Multiple kinases [50]	Yes [50]	Unresectable hepatocellular carcinoma; Advanced renal cell carcinoma; Locally recurrent or metastatic, progressive, differentiated thyroid carcinoma refractory to radioactive iodine treatment [51,52]
Anti-xCT vaccines [36,53,54,55,56]	Not investigated	Yes [36,53,54,55,56]	None

NF-kB = nuclear factor kB; VDAC = voltage-dependent anion channel.

## Data Availability

No new data were created or analyzed in this study. Data sharing is not applicable to this article.
